# Prevalence and Correlates of Erectile Dysfunction among Primary Care Clinic Attendees in Nigeria

**DOI:** 10.5539/gjhs.v4n4p107

**Published:** 2012-06-08

**Authors:** Lawrence A. Adebusoye, Olubunmi E. Olapade-olaopa, Modupe M. Ladipo, Eme T. Owoaje

**Affiliations:** 1 General Outpatients Department, University College Hospital, Ibadan, Nigeria; 2 Department of Surgery, College of Medicine, University of Ibadan, Nigeria; 3 Department of Community Medicine, College of Medicine, University of Ibadan, Nigeria

**Keywords:** correlates, erectile dysfunction, Ibadan, Nigeria, prevalence

## Abstract

**Introduction:**

Erectile dysfunction (ED) has become a public health issue in Nigeria because of its increasing magnitude, association with chronic medical conditions and negative impact on sexual life.

**Materials and Methods:**

Cross-sectional study of 450 male patients aged 18-70years who presented with non-ED related complaints. Main outcome measurements were prevalence and severity of ED which was assessed with International Index of Erectile Function (IIEF-5) and single-item sexual function questionnaire. Also assessed were socio-demographic characteristics, physical activities, sexual satisfaction and morbidities.

**Results:**

The prevalence of ED was 55.1% (mild, moderate and severe were 32.6%, 17.8% and 4.7% respectively). Prevalence of ED was significantly associated with age (p < 0.0001), marital status (p = 0.032), income (p = 0.001), social class (p = 0.004), physical activities (p = 0.006) and BMI (p = 0.012). Prevalence of ED was significantly high among men with diabetes mellitus (72.7%), hypertension (70.7%), peptic ulcer disease (70.4%) and previous prostate surgery (76.2%). Logistic regression showed dissatisfaction with sexual life (OR = 0.689, CI = 1.233-5.866; p = 0.013) and having sexual activities less than desired (OR = 3.331, CI = 1.416-7.839; p = 0.006) to be the most significant factors associated with ED. There was a strong positive correlation between the IIEF-5 and single-item sexual function questionnaire (r = 0.747, p < 0.0001).

**Conclusion:**

The prevalence of ED is high among males attending a primary care clinic in Nigeria with non-ED related complaints. ED was more prevalent in men with chronic medical illnesses and sedentary lifestyle. Family physicians should inquire about this condition in these men and refer them early for specialist consultation.

## 1. Introduction

Erectile dysfunction (ED) is defined as the inability of the male to achieve an erect penis for sufficient sexual intercourse as part of an overall multifaceted process of male sexual function. This definition takes a variety of physical aspects with important psychological and behavioural overtones into consideration (National Institute of Health [NIH], 1993; Segraves, 2010).

The prevalence of ED varies with the age, race, ethnicity, socio-economic and the health status of affected individuals (Tan, Hong, Png, Liew, & Wong, 2003). Globally, the prevalence of ED is high and is expected to increase substantially over the next 25 years (NIH, 1993). In Nigeria, the overall prevalence of the condition is between 43.8% (Fatusi, Ijaduola, & Ojofeitimi, 2003) and 57.4% (Shaeer, Osegbe, Siddiqui, Glasser, & Jaguste, 2003) as reported by studies done in 2002 and 2003 respectively. Despite these high prevalence rates, only 11.6 to 22.2% of men with ED seek medical advice, with 36.9% of these men taking their medication regularly (Costa, Avances, & Wagner, 2003).

The aetiology of erectile dysfunction could be psychological, organic, or of mixed aetiology with both factors present (NIH, 1993). The psychological causes include sexual performance anxiety, depression, bereavement, tiredness and stress (NIH, 1993; Segraves, 2010). The organic causes are common in older men with chronic diseases like diabetes mellitus, hypertension and arteriosclerosis (Elbendary, El-Gamal, & Salem, 2009).

Shyness, ignorance and reluctance to confide private matters to their physicians often prevent most couple from seeking medical help (Laumann, Glasser, Neves, & Moreira, 2009). Generally ED is considered a benign condition. However, it has significant effects on the quality of sexual life of both the patients and their partners leading to marital discord and even marital violence (NIH, 1993; Balon, 2008). This is because sexual function had been shown to be a high priority for men and their partners throughout their life span (Dunn, 2004). Loss of sexual harmony reduces the quality of life of men and their partners (Fatusi et al., 2003).

ED is thus an important public health problem with a high misconception rate (Fatusi et al., 2003). Most studies on the prevalence of ED in Nigeria were community based. Since chronic medical illnesses are becoming increasingly prevalent in our setting, a clinic based study of ED among men who have demonstrated a health-seeking behaviour and present with non-ED related problems would help physicians appreciate the magnitude and correlates of ED.

## 2. Materials and Methods

### 2.1 Study Site

This study was carried out in the General Outpatients (G.O.P) Clinic of the University College Hospital (UCH), Ibadan, the capital city of Oyo state, Nigeria. The G.O.P. clinic functions as the primary care clinic within a tertiary hospital (UCH) and is the gateway to most of the patients presenting at UCH. All patients are initially triaged and most are managed by the family physicians. Those needing specialist care are referred to the speciality units within the facility.

### 2.2 Study Design

This was a cross-sectional (descriptive) study of 450 male respondents aged 18 to 70years who presented between February 2005 and April 2005 at the G.O.P. clinic, UCH.

### 2.3 Ethical Consideration

Approval for the study was obtained from the Head of the G.O.P Department, UCH, Ibadan, and the University of Ibadan/University College Hospital Institutional Ethical Review Board (UI/UCH IRB). Approval number: UI/IRC/04/0112.

### 2.4 Sampling Technique

This was done using systematic sampling technique. Averagely, 1243 new adult patients are seen monthly at the G.O.P clinic, with 474 (38.1%) of them being males. During the 3 months period of the study, one in every three patients seen at the clinic was recruited. Those presenting primarily with ED were excluded from the study. Informed consent of each respondent was obtained before examination and administration of questionnaire.

### 2.5 Procedure

The respondents were interviewed using the IIEF-5 questionnaire which had been validated and accepted by the Food and Drug Administration (FDA) in the United States of America for use in ED trials.^11^ IIEF-5 has also been used in some Nigerian studies (Fatusi et al., 2003; Shaeer et al., 2003). The IIEF-5 has been adopted as the “Gold standard” measure for efficacy assessment in clinical trials of ED (Johannes et al., 2000; Rosen, Cappelleri, & Gendrano, 2002). The score for the IIEF ranges from 0 to 25. Erectile function is classified into four classes based on these scores (Rosen et al., 2002); Severe ED 0–7, moderate ED 8–16, mild ED 17–21 and normal 22–25.

The respondents’ socio-demographic characteristics which included their marital status, income and social class were obtained. Detailed history was taken, which included the frequency of sexual activity and early morning erection, level of physical activity, current medications and psychological health. Additional information on the respondents’ satisfaction with their sexual life, satisfaction with their partner(s) and their partner(s) satisfaction with their sexual relationship was obtained using a likert scale from 1= extremely satisfied to 5 = extremely dissatisfied. Physical examination including anthropometric measurement, and laboratory investigations were done as necessary to arrive at the diagnoses.

In addition, a single structured questionnaire derived directly from the widely accepted National Institute of Health (NIH) consensus on ED, which had been used in many Cross-National studies (including those done in Nigeria) on ED was also used to assess the sexual performance of the respondents (NIH, 1993; Shaeer et al., 2003).

### 2.6 Anthropometric Measurements

Height was recorded in metres with a measurement stand (stadiometer) and was measured to the nearest 0.1 centimetre. Weight was measured with a weighing scale manufactured by Hana, China and recorded to the nearest 0.1 kilogram. The body mass index (BMI) was calculated from the weight in kilogrammes divided by height in meters squared (World Health Organization [WHO], 1995). This was graded as underweight BMI < 18.4kg/m^2^, normal (BMI 18.5 – 24.9 kg/m^2^), overweight (BMI of 25.0 – 29.9 kg/m^2^) and obesity (BMI > 30.0 kg/m^2^) (WHO, 1995).

Blood pressure was measured with a mercury sphygmomanometer made by Accosson^R^ England, which was calibrated and validated before use. Respondents were allowed to rest, and measurement started after 5 minutes. Appropriate sized cuffs were used for each patient, encircling at least 80% of the arm. The average of two readings taken 5 minutes apart was recorded as the blood pressure (World Health Organization International Society of Hypertension writing group [WHO-ISH], 1999). The staging of hypertension was done according to the seventh report of the Joint National Committee on prevention, detection, evaluation and treatment of Hypertension (Joint National Committee on prevention, detection, evaluation and treatment of Hypertension seventh report [JNC-7], 2003). Stage 1 hypertension was taken as systolic blood pressure of 140 to 159mmHg and diastolic blood pressure of 90 to 99mmHg, while stage 2 was any systolic blood pressure of greater than 160mmHg and diastolic blood pressure greater than 100mmHg (JNC-7, 2003).

The respondents’ social class was class defined according to the British Registrar-general’s classification (Rose, 1995). Class I (Professionals) was allocated to lawyers, doctors, accountants, and like professionals; Class II (Intermediate) to senior public servants, teachers, nurse, and managers; Class III (Skilled non-manual) to typists, shop assistants, and artisans; Class IV (Partly skilled manual) to farm-workers, drivers and bus-conductors; and Class V (Unskilled manual) to housewives, petty traders, cleaners, labourers.

### 2.7 Data Analysis

The administered questionnaires were collated and cross-checked after each interview and coded serially. Data entering, cleaning and analysis were done using SSPS (version 15). Descriptive statistics was used to describe socio-demographic characteristics of the respondents. Chi-square statistics was used to assess association between categorical variables. Logistic regression analysis was used to explore relationship between socio-demographic characteristics, sexual activities and ED. Values of significance was set at p < 0.05.

## 3. Results

Four hundred and fifty adult male respondents aged 18 to 70 years were studied. The median age (SD) was 39.0 (±15.8) years. Majority of respondents (68.2%) were married, while 28.9% were single and had regular sexual partners. The median monthly income (SD) was 10,000 (Interquartile range 4,000 to 125,000) Naira ($26.67 to $833.33). The frequency of sexual activities by respondents per week was 1.58 ± 1.04 times (0 – 6 times per week). The median early morning erection achieved by respondents was 3.0 ± 1.95 days per week.

**Figure 1:**
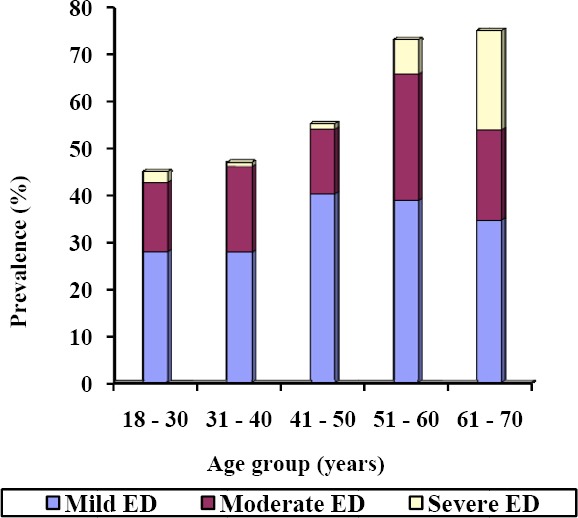
Severity of erectile dysfunction by age

The median score of respondents on IIEF-5 was 20.0 (Interquartile range 14.0 to 23.0). The prevalence of ED was 55.1%; with 147(32.6%), 80(17.8%) and 21(4.7%) respondents having mild, moderate and severe ED respectively. The prevalence of the ED increased with age from 45.0% in the age group 18 to 30 years, through 47.0% in the age group 31 – 40 years, and 55.2% in the age group 41 – 50 years, to 73.1% and 75.0% in the age groups 51 – 60 and 61 – 70 years respectively (Figure 1).

Using the single-item sexual function questionnaire, 222 (49.3%) of respondents were not always able to initiate and maintain an erection good enough for satisfactory intercourse, which signifies erectile dysfunction. One hundred and fourteen (25.3%) respondents were usually able to initiate and maintain an erection good enough for satisfactory intercourse (mild ED), 83(18.5%) were sometimes able to initiate and maintain an erection good enough for satisfactory intercourse (moderate ED) and 31(6.9%) were completely unable to initiate and maintain an erection good enough for satisfactory intercourse (severe ED). There was a strong positive correlation between the IIEF-5 and the single-item sexual function questionnaire (r = 0.747, p < 0.0001)

Table 1 showed the socio-demographic characteristics of respondents by the prevalence of ED. The prevalence of ED increased significantly from 45.0% in the age group 18-30 years to 75.0% in the 61-70 years age group. There was a statistical association between increasing age and the prevalence of ED (χ^2^ = 25.576, p < 0.0001). Among respondents who were currently married, the prevalence of ED was 57.3%. This was higher than the prevalence among respondents who were single (46.9%), but was lower than the prevalence among respondents who were widowed (85.7%) and separated or divorced (83.3%). The prevalence of ED was observed to increase with social class from 52.2% in social class V to 90.0% in social class I except for a slight decrease among respondents in social class IV (45.7%). There was a statistical association between the prevalence of ED and increase in social class (χ^2^ = 15.490, p = 0.004). Using the World Bank’s poverty line of $1 a day (World Bank, 2004), higher proportion of respondents living above poverty line had ED compared with those living below poverty line (57.6% vs 40.9%) (χ^2^ = 6.306, p = 0012).

**Table 1:** Socio-demographic characteristics of respondents

	ED = 248, n (%)	No ED =202, n (%)	Total= 450, N (%)
**Age groups (years)**			
18 – 30	58 (45.0)	71 (55.0)	129 (100.0)
31 – 40	54 (47.0)	61 (53.0)	115 (100.0)
41 – 50	48 (55.2)	39 (44.8)	87 (100.0)
51 – 60	49 (73.1)	18 (26.9)	67 (100.0)
61 – 70	39 (75.0)	13 (25.0)	52 (100.0)
χ^2^= 25.576, df = 4; p < 0.0001*.			
**Marital status**			
Married	176 (57.3)	131 (42.7)	307 (100.0)
Single	61 (46.9)	69 (53.1)	130 (100.0)
Separated or Divorced	5 (83.3)	1 (16.7)	6 (100.0)
Widowed	6 (85.7)	1 (14.3)	7 (100.0)
χ^2^=8.715, df=3; Fisher’s p=0.032*			
**Income**			
Earned above $1 a day	221 (57.6)	163 (42.4)	384 (100.0)
Earned below $1 a day	27 (40.9)	39 (59.1)	66 (100.0)
χ^2^= 6.306, df = 1; p = 0.001*			
**Social class**			
I	9 (90.0)	1 (10.0)	10 (100.0)
II	54 (67.5)	26 (32.5)	80 (100.0)
III	20 (64.5)	11 (35.5)	31 (100.0)
IV	48 (45.7)	57 (54.3)	105 (100.0)
V	117 (52.2)	107 (47.8)	224 (100.0)
χ^2^= 15.490, df = 4; p = 0.004*			

* Significant at 5% level of significance

Table 2 describes the lifestyle habits and physical characteristics of respondents. Higher proportion of men who were smokers had ED when compared with those who had either quit or never smoked tobacco (57.7% vs 55.0%, p = 0.963). One hundred and two respondents drank alcohol regularly, of these 48 (47.1%) had ED. All the four men using cannabis in this study had ED. Higher proportion of respondents who drink coffee regularly had ED compared with those who had never drank coffee or quit taken coffee (61.0% vs 54.5%, p = 0.705). The prevalence of ED increased significantly as the level of physical activities decreased from 47.7% in respondents who were very active to 70.7% in those who were not active (χ^2^ = 10.296, p = 0.006). Fifty-three percent of respondents who had normal BMI had ED, while 40.7% of underweight respondents had ED. On the other hand, 71.3% of respondents who were overweight and 46.7% of those who were obese had ED. There was a significant association between the overall prevalence of ED and increase in BMI χ^2^ = 13.173, p = 0.004).

**Table 2:** Lifestyle and physical characteristics

	ED = 248, n (%)	No ED =202, n (%)	Total =450, N (%)
**Alcohol intake**			
Yes	48 (47.1)	54 (52.9)	102 (100.0)
No	120 (57.7)	88 (42.3)	208 (100.0)
Quit	80 (57.2)	60 (42.8)	140 (100.0)
χ^2^= 3.467, df = 2; p = 0.177			
**Tobacco intake**			
Yes	15 (57.7)	11 (42.3)	26 (100.0)
No	179 (54.9)	147 (45.1)	326 (100.0)
Quit	54 (55.1)	44 (44.9)	98 (100.0)
χ^2^= 0.075, df = 2; p = 0.963			
**Cannabis use**			
Yes	4 (50.0)	0 (0.0)	4 (100.0)
No	244 (54.7)	202 (45.3)	446 (100.0)
χ^2^= 1.711, df = 1; p = 0.191 (Yates’s corrected)			
**Coffee use**			
Yes	25 (61.0)	16 (39.0)	41 (100.0)
No	216(54.4)	181 (45.6)	397 (100.0)
Quit	7 (58.3)	5 (41.7)	12 (100.0)
χ^2^= 0.700, df = 2; p = 0.705			
**Physical activities**			
Not active	53 70.7)	22 (29.3)	75 (100.0)
Moderately active	133 (54.3)	112 (45.7)	245 (100.0)
Very active	62 (47.7)	68 (52.3)	130 (100.0)
χ^2^= 10.296, df = 2; p = 0.006*			
**BMI classification**			
Underweight	13 (40.6)	19 (59.4)	32 (100.0)
Normal	166 52.5)	150 (47.5)	316 (100.0)
Overweight	62 (71.3)	25 (28.7)	87 (100.0)
Obese	7 (46.7)	8 (53.3)	15 (100.0)
χ^2^= 13.173, df = 3; p = 0.004*			

* Significant at 5% level of significance

Table 3 showed significant association between the prevalence of ED and hypertension (70.7%, p = 0.011), diabetes mellitus (72.7%, p = 0.001), peptic ulcer disease (70.4%, p < 0.0001) and previous prostate surgery (76.2%, p = 0.047). However, there was no statistical association between prevalence of ED and depression (59.2%, p = 0.263). Majority of men who were using antihypertensive drugs (57.1%), antidepressants (83.3%), peptic ulcer drugs (50.0%) and oral hypoglycemic agents (87.5%) had ED. However, there was no statistical association between these medications and the prevalence of ED.

**Table 3:** Clinical correlates of erectile dysfunction

	ED = 248 n (%)	No ED = 202 n (%)	Total = 450 N (%)	χ^2^	p–value
**Medical illnesses**					
Hypertension	41 (70.7)	17 (29.3)	58 (100.0)	6.532	0.011*
Diabetes mellitus	24 (72.7)	9 (27.3)	33 (100.0)	10.830	0.001*
Peptic ulcer Disease	38 (70.4)	16 (29.6)	54 (100.0)	15.455	<0.0001*
Depression	77 (59.2)	53 (40.8)	130(100.0)	1.254	0.263
Prostate surgery	16 (76.2)	5 (23.8)	21 (100.0)	3.957	0.047*
**Medications**					
Antihypertensives	12 (57.1)	9 (42.9)	21(100.0)	0.037	0.848
Antidepressants	5 (83.3)	1 (16.7)	6 (100.0)	0.972 (Yates’s corrected)	0.324
Peptic ulcer drugs	4 (50.0)	4 (50.0)	8 (100.0)	0.000 (Yates’s corrected)	1.000
Oral hypoglycemic agents	7 (87.5)	1 (12.5)	8 (100.0)	2.249(Yates’s corrected)	0.134

* Significant at 5% level of significance

Sexual life and partner(s)’ satisfaction is shown in Table 4. The prevalence of ED increased significantly with the level of dissatisfaction with respondents’ sexual life from 36.1% in those who were extremely satisfied to 100.0% in those who were extremely dissatisfied (χ^2^ = 80.039 p < 0.0001). Similarly, the prevalence of ED increased significantly with the respondents’ dissatisfaction with their sexual relationship with present partner(s); 42.0% of respondents with ED were extremely satisfied, while 80.0% were extremely dissatisfied (χ^2^ = 65.599, p < 0.0001). There was a significant association between the prevalence of ED and the level of dissatisfaction the partner(s) sexual relationship (χ^2^ = 41.418, p < 0.0001).

**Table 4:** Sexual life and partner(s)’ satisfaction (N=450)

	Extremely satisfied	Somewhat satisfied	Neither satisfied nor dissatisfied	Somewhat dissatisfied	Extremely dissatisfied
**How satisfied are you with your sexual life?**					
Have ED (%)	68 (36.1)	77 (53.1)	62 (84.9)	24 (88.9)	17 (100.0)
Have no ED (%)	120 (63.9)	68 (46.9)	11(15.1)	3 (11.1)	0 (0.0)
Total (%)	188(100.0)	145(100.0)	73 (100.0)	27 (100.0)	17 (100.0)
χ^2^= 80.039 df = 4 p < 0.0001*					
**How satisfied are you with your sexual relationship with present partner(s)?**					
Have ED (%)	86 (42.0)	64 (48.1)	62 (86.1)	24 (96.0)	12 (80.0)
Have no ED (%)	119 (58.0)	69 (51.9)	10 (13.9)	1 (4.0)	3 (20.0)
Total (%)	205(100.0)	133(100.0)	72 (100.0)	25 (100.0)	15 (100.0)
χ^2^= 65.599 df = 4 p < 0.0001*					
**How satisfied do you think your partner(s) are with your sexual relationship?**					
Have ED (%)	79 (44.9)	75 (47.8)	61 (78.2)	18 (85.7)	15 (83.3)
Have no ED (%)	97 (55.1)	82 (52.2)	17 (21.8)	3 (14.3)	3 (16.7)
Total (%)	176(100.0)	157(100.0)	78 (100.0)	21 (100.0)	18 (100.0)
χ^2^= 41.418 df = 4 p < 0.0001*					

* Significant at 5% level of significance

The desire for sexual activities with the partner(s) is shown in Table 5. Majority of respondents 80.9% who had sexual activities less than they desired had ED as compared with 41.5% of respondents who had sexual activities as much as they desire. There was a significant inverse association between the desire for sexual activities and prevalence of ED (χ^2^ = 63.597, p < 0.0001).

**Table 5:** Sexual activities with the partner(s)

Desire for sexual activities	ED n=248(%)	No ED n=202(%)	Total N = 450(%)
Less than i desired	123 (80.9)	29 (19.1)	152 (100.0)
As much as i desired	122 (41.5)	172 (58.5)	294 (100.0)
More than i desired	3 (75.0)	1 (25.0)	4 (100.0)
χ^2^= 63.597 df = 2 p < 0.0001*			

Logistic regression was done using all the variables that showed a significant association with erectile dysfunction. Satisfaction with sexual life (OR = 2.689, CI = 1.233 – 5.866; p = 0.013) and desire for sexual activities with the partner(s) (OR = 3.331, CI = 1.416 – 7.839; p = 0.006) were found to be the most significant factors associated with ED (Table 6).

**Table 6:** Logistic regression analysis

	β	SE	Wald	df	p-value	Odds Ratio	Confidence Interval
**Age**	0.279	0.193	2.088	1	0.148	1.322	0.905 – 1.930
**Marital status**	0.011	0.282	0.002	1	0.968	1.012	0.582 – 1.757
**Religion**	-0.425	0.344	1.529	1	0.216	0.654	0.333 – 1.282
**Occupation**	1.254	1.256	0.996	1	0.318	3.504	0.299 – 7.175
**Satisfaction with sexual life**	0.989	0.398	6.180	1	0.013*	2.689	1.233 – 5.866
**Satisfaction with sexual relationship with your present partner(s)**	0.069	0.436	0.025	1	0.874	1.072	0.456 – 2.518
**Partner(s) satisfaction with your sexual relationship**	-0.447	0.307	2.117	1	0.146	0.639	0.350 – 1.168
**Desire for sexual activity**	1.203	0.437	7.596	1	0.006*	3.331	1.416 – 7.839
**Constant**	-2.682	0.903	8.833	1	0.003		

* Significant at 5% level of significance

## 4. Discussion

ED is a major cause of sexual and family problems globally as its prevalence is high and is still increasing. ED causes loss of sexual satisfaction with its attendant negative impact on the psychological well-being, social health and the family relationship of the sufferers.

Globally, the prevalence of ED varies with race, ethnicity, socioeconomic and health status of the affected individuals (NIH, 1993). Men of African descent have been found to have greater risk of developing ED than the Caucasians (Laumann & Rosen, 2007). They had different lifestyle habits and health conditions compared with Caucasians as they are poorer, and have more psychosocial problems, particularly depression, anxiety and poor partner relationships (Laumann & Rosen, 2007). The overall prevalence of ED in this study was 55.1% with about half of the respondents having mild ED. Using similar tool (the IIEF), the overall prevalence of ED in our study is higher than those from Asian countries like China (28.3%) (Quan et al., 2004); South Korean (36.6%) (Cho, Kim, & Choi, 2003); and Thailand (42.2%) (Permpongkosol, Kongkakand, Ratana-Olarn, Tantiwong & Tantiwongse, 2008). Similarly, it was greater than the prevalence of ED in Holland (17%) (De Boer, 2004); Austria (32.2%) (Ponholzera et al., 2005) and Canada (49.4%) (Grover et al., 2006).

Age was found to be a significant factor to developing ED in this study. Its severity was also found to worsen with increasing age. This pattern was reported in virtually all the studies on ED (NIH, 1993, Fatusi et al., 2003; Shaeer et al., 2003). The prevalence of chronic medical illnesses like hypertension, diabetes mellitus, and depression which increase with age also contributes to this phenomenon (Shaeer et al., 2003).

Most studies on ED had shown that the prevalence of ED was high in men having chronic medical illnesses as well as those on medications for these illnesses (Segraves, 2010; Elbendary et al., 2009; Berada, Kadri, Mechakra-Tahiri, & Nejjari, 2003). About one in every five cases of ED had been attributed to adverse drug events mostly from chronic use of antihypertensive drugs especially older ones like thiazides, β-blockers and centrally acting antihypertensives (Doumas & Doumas, 2006). However, newer antihypertensive drugs like calcium channel blockers, angiotensin-converting enzyme (ACE) inhibitors, and angiotensin receptor blockers (ARBs) have been found to have neutral effects or may even be beneficial with respect to erectile function (Doumas & Doumas, 2006). Vascular diseases had been reported in literature as the cause of nearly half of all cases of ED in men older than 50 years (Berada et al., 2003; Montorsi, Ravagnani, & Galli, 2009). Similar study among Canadians attending family practice clinics showed cardiovascular disease to be an independent risk factor with ED (Grover et al., 2006). The aetiology of ED in vascular diseases is the impairment of endothelial function, which occurs in arteriosclerosis, peripheral vascular disease, myocardial infarction, and arterial hypertension (Montorsi et al., 2009). Hypertension and diabetes mellitus affect the neurovascular axis of the erectile pathway, while depression affects the higher centres of the brain initiating sexual arousal and behaviour (Shaeer et al., 2003; Elbendary et al., 2009). However, the possible pathway of peptic ulcer disease in the aetiology of ED is not clear, and has been the subject of much debate (Berada et al., 2003). Peptic ulcer disease is however associated with stress, tobacco smoking, excessive alcohol consumption; and medications like cimetidine used in its treatment all of which could cause ED (Berada et al., 2003; Elbendary et al., 2009). There was a significant association between ED and previous prostate surgery in our study (p = 0.007). The surgical methods commonly used for prostate surgery like retropubic prostatectomy (RPP) and transvesical prostatectomy (TVP) and trans-urethral resection which are done in our environment could predispose to injuries to the pelvic nerves necessary for erection. Newer surgical techniques for prostate surgery like laser, microwave and radio frequency ablation which are practiced in the developed countries had been found to be rarely associated with ED (Incrocci, Hop, & Slob, 2003).

The prevalence of ED in this study increased significantly with the level of social class. This was in contrast to the findings among Moroccan and Korean men where an inverse relationship between the social class and the prevalence of ED was reported (Cho et al., 2003; Berrada, 2003). The prevalence of ED inversely correlated with the level of physical activities. Many studies had shown that engaging in physical activity is an important life style modification, which is beneficial in the management of risk factors to ED like hypertension, obesity and diabetes mellitus (NIH, 1993; Shaeer et al., 2003; Elbendary et al., 2009). Additionally, it improves cardiovascular status, self-esteem and overall health which reduce the risk of ED.

Good sexual function had been shown to be a high priority for men and their partners throughout their life span and the loss of sexual harmony had been found to reduce the sexual satisfaction and quality of life of men and their partners (NIH, 1993; Balon, 2008). Our study showed significant association between the severity of ED and the respondents’ dissatisfaction with sexual life, respondents’ dissatisfaction with their partners and partners’ dissatisfaction with the respondents. Respondents who had no ED were found to be mostly satisfied with their sexual life and satisfied with their partners. They also believed that their partners were satisfied with them. Men with ED were less content with their (sexual) life and had less confidence in sexual performance (De Boer et al. 2004). Presence of ED was negatively related to affected happiness in life (De Boer et al., 2004). Berrada et al., (2003) reported similar findings of significant reduction in the level of satisfaction with sexual life and satisfaction with partners as well as the partner’s satisfaction, with increasing severity of ED among Moroccan men. The high prevalence of ED we observed among men who were either divorced or separated was therefore not surprising since ED is known to cause strained relationship and significant marital difficulties (NIH, 1993; Elbendary et al., 2009). Shaeer et al. (2003) reported similar findings in the three-nation survey on ED.

There was a significant inverse association between the frequency of sexual activities and the prevalence of ED (p < 0.0001). This corroborated the Moroccan study which found 39% and 100% of men with moderate and severe ED having no sexual activities in the previous 6months compared with 9.4% and 10.3% of those with no ED and mild ED reporting no sexual activities (Berrada et al., 2003). Reduced frequency of sexual activities is associated with stress in interactions with one’s sexual partner leading to marital discord and even marital violence (NIH, 1993; Balon, 2008). Respondents who were not satisfied with their sexual life had 2.7 times risk of having ED, while those whose frequency of desired sexual activities with the partner(s) were less than desired had 3.3 times the risk of having ED.

ED is a problem associated with a lot myths and misconception in Nigeria. Among married Nigerian men, Fatusi et al. (2003) reported that 38.9% believed ED to be a myth, and another 23.6% attributed it to various causes other than being a natural aging process. Discussing sexual issues is difficult for the sufferers and their physicians. Less than half of Nigerian men (40.9%) could discuss sex freely with anybody including their spouse and physicians (Okonkwo, Uwakwe, Obionu, & Okonkwo, 2010). Also, most of the primary care physicians in Nigeria (62%) would not take sexual history unless patient brought it up (Ariba, Oladapo, Iyaniwura, & Dada, 2007). The reported barriers to the management of ED in our setting include lack of a standardised protocol, inadequate experience in ED management, preference of patients for native medication, and the high cost of modern medication (Ariba et al., 2007). Similarly, Pertula, 1999 reported that only 27% of physicians at a family practice clinic in USA ask all male patients they see about their sexual function. This was quite surprising, as over a half (56.2%) of primary care physicians in Nigeria ascribed a high priority to ED management in their day-to-day practice and believed that four-fifth of ED sufferers could benefit from orthodox treatment, however, only 18% of them had ever prescribed any medications for affected sufferers (Ariba et al., 2007).

Lengthy questionnaire could be a hindrance to routine evaluation of sexual function by physicians. Since there was a strong positive correlation between the IIEF-5 and single-item sexual questionnaire (r = 0.747, p < 0.0001) in this study, having the single-item sexual function questionnaire especially in the primary care setting would afford assessment of ED within the constrain of routine clinical consultation.

## 5. Conclusion

High prevalence of ED in this study underscored the need for routine sexual evaluation of all men presenting to the physicians in our setting so as to diagnose ED early. Increasing age was found to be a major predisposing factor to developing ED, but older men should not be denied the benefit of proper management of the disease. The presence of ED could be a pointer to underlying medical illnesses, and this should prompt a thorough medical screening of the patient. Careful prescription of medications to treat chronic medical illnesses is also necessary in order not to precipitate or worsen ED. Patients attending primary care should also be educated on lifestyle modification and habits, since these have been shown to be significantly associated with ED. Family in Physicians should refer men diagnosed with ED early for specialist management to minimise morbidity.

## References

[ref1] Ariba A. J, Oladapo O. T, Iyaniwura C. A, Dada O. A (2007). Management of erectile dysfunction: perceptions and practices of Nigerian primary care clinicians. SA Fam. Pract.

[ref2] Bai Q, Xu Q-Q, Jiang H, Zhang W-L, Wang X-H, Zhu J-C (2004). Prevalence and risk factors of erectile dysfunction in three cities of China: a community-based study. Asian J. Androl.

[ref3] Balon R (2008). Sexual Dysfunction. The Brain-Body Connection. Adv. Psychosom Med. Basel.

[ref4] Berrada S, Kadri S, Mechakra-Tahiri S, Nejjari C (2003). Prevalence of erectile dysfunction and its correlates: a population-based study in Morocco. International Journal of Impotence Research.

[ref5] Cho B. L, Kim Y. S, Choi Y. S (2003). Prevalence and risk factors for erectile dysfunction in primary care: results of a Korean study. International Journal of Impotence Research.

[ref6] Costa P, Avances C, Wagner L (2003). Erectile dysfunction: Knowledge, wishes and attitude. Results of a French study of 5,099 men aged 17–70 years. Prog. Urol.

[ref7] Boer B. J, DeBots M. L, Lycklama a Nijeholt A. A, Moors J. P, Pieters H. M, Verheij T. M (2004). Erectile dysfunction in primary care: prevalence and patient characteristics. The ENIGMA study. International Journal of Impotence Research.

[ref8] Doumas M, Doumas S (2006). The Effect of Antihypertensive Drugs on Erectile Function: A Proposed Management Algorithm. J. Clin. Hypertens.

[ref9] Dunn M. E (2004). Restoration of couple’s intimacy and relationship vital to re-establishing erectile function. J. Am. Osteopath. Assoc.

[ref10] Elbendary M, El-Gamal O, Salem K (2009). Analysis of Risk Factors for Organic Erectile Dysfunction in Egyptian Patients Under the Age of 40 Years. Journal of Andrology.

[ref11] Fatusi A. O, Ijaduola K. T, Ojofeitimi E. O (2003). Assessment of andropause awareness and erectile dysfunction among men in Ile-ife, Nigeria. Aging male.

[ref12] Grover S. A, Lowensteyn I, Kaouache M, Marchand S, Coupal L, DeCarolis E, Defoy I (2006). The Prevalence of Erectile Dysfunction in the Primary Care Setting: Importance of Risk Factors for Diabetes and Vascular Disease. Arch. Intern. Med.

[ref13] Incrocci L, Hop W. C, Slob A. K (2003, July). Efficacy of sildenafil in an open-label study as a continuation of a double-blind study in the treatment of erectile dysfunction after radiotherapy for prostate cancer. Urology.

[ref14] Johannes C. B, Araujo A. B, Feldman H. A, Derby C. A, Kleinman K. P, McKinlay J. B (2000). Incidence of erectile dysfunction in men 40 – 69 years old: Longitudinal results from Massachusetts Male Aging Study. J. Urol.

[ref15] Joint National Committee on Prevention, Detection, Evaluation, and Treatment of High Blood Pressure (2003). The Seventh Report of the Joint National Committee on Prevention, Detection, Evaluation, and Treatment of High Blood Pressure. The JNC- 7 Report [document on the Internet]. Department of Health and Human Services.

[ref16] Laumann E. O, Glasser D. B, Neves R. C, Moreira E. D (2009). GSSAB Investigators’ Group. A population-based survey of sexual activity, sexual problems and associated help-seeking behavior patterns in mature adults in the United States of America. Int. J. Impot. Res.

[ref17] Laumann E, Rosen R (2007, February). Erectile dysfunction influenced by race and ethnicity. Science Daily.

[ref18] Montorsi P, Ravagnani P. M, Galli S (2009). The Triad of Endothelial Dysfunction, Cardiovascular Disease and Erectile Dysfunction: Clinical Implications. European Urology Supplements.

[ref19] National Institute of Health (1993). Consensus development panel on impotence. NIH Consensus conference. Impotence. JAMA.

[ref20] Okonkwo J. E. N, Uwakwe R, Obionu C, Okonkwo C. V (2010). Communication and sexuality in a Nigerian community. Advances in Conception.

[ref21] Permpongkosol S, Kongkakand A, Ratana-Olarn K, Tantiwong A, Tantiwongse K (2008). Increased prevalence of erectile dysfunction (ED): results of the second epidemiological study on sexual activity and prevalence of ED in Thai males. Aging Male.

[ref22] Perttula E (1999, February). Physician attitude and behaviour regarding erectile dysfunction in at-risk patients from a rural community. Postgraduate Med J.

[ref23] Ponholzera A, Temmlb C, Mocka K, Marszaleka M, Obermayrc R, Madersbachera S (2005). Prevalence and Risk Factors for Erectile Dysfunction in 2869 men using a validated questionnaire. European Urology.

[ref24] Rose D (2005). Official social classifications in the UK. Social Research Update. [serial on the Internet].

[ref25] Rosen R. C, Cappalleri J. C, Gendrano N (2002). The International Index of Erectile Dysfunction (IIEF-5): A State-of-the-science review. Int. J. of Impotence Research.

[ref26] Segraves R. T (2010). Considerations for Diagnostic Criteria for Erectile Dysfunction in DSM-V. J. Sex Med.

[ref27] Shaeer K. Z. M, Osegbe D. N, Siddiqui S. H, Glasser D. B, Jaguste V (2003). Prevalence of erectile dysfunction and its correlates among men attending primary care clinics in three Countries: Pakistan, Egypt and Nigeria. International Journal of Impotence Research.

[ref28] Tan J. K, Hong C. Y, Png D. J, Liew L. C, Wong M. L (2003). Erectile Dysfunction in Singapore: Prevalence and Its Associated Factors – A Population-Based Study. Singapore Med. J.

[ref29] World Bank (2004). Global poverty down by half since 1981 but progress uneven as economic growth eludes many countries. Press release.

[ref30] World Health Organization (1995). Physical Status: the Use and Interpretation of Anthropometry. Technical Report Series, no. 854.

[ref31] World Health Organization–International Society of Hypertension (1999). WHO–ISH guidelines for the management of hypertension. J. Hypertens.

